# Supplementary material for: Haloalkalitolerant Fungi from Sediments of the Big Tambukan Saline Lake (Northern Caucasus): Diversity and Antimicrobial Potential
Source: Microorganisms. 2023 Oct 19;11(10):2587. doi: 10.3390/microorganisms11102587 (PMC10609068; doi:10.3390/microorganisms11102587)
Supplement: Supplementary file 1 [file microorganisms-11-02587-s001.zip › microorganisms-2634506-supplementary.pdf]

Supplementary

Table S1. Sequences fungal species used in this study with their GenBank accession numbers

Species	Isolate No.	GenBank accession No.	
		ITS	beta-tubulin
Acremonium alternatum Link	CBS 407.66 T	NR_144913.1	-
Acremonium egyptiacum (J.F.H. Beyma) W. Gams	CBS 114785 T	NR_163807.1	-
	A130	KC987166	-
	A101	KC987139	-
	CBS 124.42	MH856101	-
	**p19 / SLF 0218.0913**	**OR335864**	-
	**p33 / SLF 0218.1001**	**OR335865**	-
Alternaria alternata (Fr.) Keissl.	CBS 916.96 T	FJ196306	-
	CBS 137513	KJ443247	-
	**p18 / SLF 0218.0604**	**OR335862**	-
	**p44 / SLF 0218.0306**	**OR335861**	-
Alternaria chlamydospora Mouch.	CBS 491.72 T	KC584189	-
Alternaria molesta E.G. Simmons	CBS 548.81 T	KC584205	-
	CBS 137524	KJ443261	-
Alternaria obclavata (Crous & U. Braun) Woudenb. & Crous	CBS 124120 T	KC584225	-
Alternaria shukurtuzi Bilanenko, Georgieva & Grum-Grzhim.	CBS 137520 T	KJ443257	-
Alternaria soliaridae E.G. Simmons	CBS 118387 T	KC584218	-
Alternaria kulundae Bilanenko, Georgieva & Grum-Grzhim.	CBS 137525 T	KJ443262	-
	CBS 137522	KJ443259	-
	CBS 137521	KJ443258	-
Alternaria petuchovskoi Bilanenko, Georgieva & Grum-Grzhim.	CBS 137517 T	KJ443254	-
Aspergillus alboluteus F. Sklenar, Jurjevič, Ezekiel, Houbraken & Hubka	CBS 145855 T	MW448663	-
Aspergillus alboviridis J.P.Z. Siqueira, GenJ.P.Z. Siqueira, Gené, F. Sklenar & Hubka	CBS 142665 T	LT798909	-
Aspergillus ardalensis A. Nováková, Hubka, Kolařík & S.W. Peterson	CBS 134372 T	FR733808	-
Aspergillus creber Jurjević, S.W. Peterson & B.W. Horn	CBS 145749 T	NR_135442	-
Aspergillus flavipes (Bainier & R. Sartory) Thom & Church	NRRL 302 T	EF669591	-
Aspergillus fumigatus Fresen.	CBS 133.61 T	EF669931	-
Aspergillus glaucus (L.) Link	CBS 516.65 T	EF652052	-
Aspergillus iizukae Sugiyama	CBS 541.69 T	OL711774.1	-
	CBS 138188 (ex-type of A. capensis)	OL711795	-
Aspergillus inusitatus F. Sklenar, C. Silva Pereira, Houbraken & Hubka	CBS 147044 T	MW448669	-
Aspergillus lanuginosus F. Sklenar & Hubka	NRRL 4610 T	EF669604	-
Aspergillus luppiae Hubka, A. Novkov, M. Kolak & S.W. Peterson	CBS 653.74 T	EF669617	-
Aspergillus micronesiensis Visagie, Hirooka & Samson	CBS 138183 T	KJ775548	-
Aspergillus movilensis A. Nováková, Hubka, Kolařík & S.W. Peterson	CBS 134395 T	HG915904	-
Aspergillus neoflavipes Hubka, A. Novkov, M. Kolak & S.W. Peterson	CBS 260.73 T	EF669614	-

Aspergillus polyporicola Hubka, A. Nováková, M. Kolarík & S.W. Peterson	NRRL 32683 T	EF669595	-
Aspergillus spelaeus A. Nováková, Hubka, M. Kolarík & S.W. Peterson	CBS 134371 T	HG915905	-
Aspergillus subversicolor Jurjević, S.W. Peterson & B.W. Horn	CBS 145751 T	JQ301894	-
Aspergillus suttoniae J.P.Z. Siqueira, Gené, Dania García & Guarro	UTHSCSA DI14-215 T	LT899487	-
Aspergillus sydowii (Bainier & Sartory) Thom & Church	CBS 593.65 T	EF652451	-
Aspergillus templicola Visagie, Hirooka & Samson	CBS 138181 T	KJ775545	-
Aspergillus urmiensis Arzanlou, Houbraken & Samadi	CBS 139558 T	KP987073	-
Aspergillus versicolor (Vuill.) Tirab.	CBS 583.65 T	EF652442	-
Aspergillus* sp. sect. *Flavipedes* ser. *Flavipedes	**p14 / SLF 0218.0503**	**OR335848**	-
Aspergillus* sp. sect. *Flavipedes* ser. *Flavipedes	**p35 / SLF 0218.0110**	**OR335851**	-
Aspergillus* sp. sect. *Flavipedes* ser. *Spelaei	**p11 / SLF 0218.0402**	**OR335847**	-
Aspergillus* sp. sect. *Flavipedes* ser. *Spelaei	**p34 / SLF 0218.0911**	**OR335850**	-
Aspergillus* sp. sect. *Nidulantes* ser. *Versicolores	**p10 / SLF 0218.0315**	**OR335846**	-
Aspergillus* sp. sect. *Nidulantes* ser. *Versicolores	**p16 / SLF 0218.0914**	**OR335849**	-
Chordomyces albus A. Giraldo, Deanna A. Sutton & Guarro	CBS 987.87 T	DQ825970	-
Chordomyces antarcticus Bilanenko, Georgieva & Grum-Grzhim.	CBS 120045 T	KJ443241	-
***Chordomyces* sp.**	**p42 / SLF 0218.0408**	**OR335860**	-
Curvularia homomorpha (Luttr. & Rogerson) Y.P. Tan & R.G. Shivas	CBS 156.60 T	JN192380	-
Emericellopsis alkalina Bilanenko & Georgieva	CBS 127350 T	KC987171	KC987133
	A117	KC987154	KC987116
	A118	KC987155	KC987117
	A119	KC987156	KC987118
	CBS 120049	KC987170	KC987132
	**p30 / SLF 0218.0608**	**OR335874**	**OR287057**
	**p36 / SLF 0218.0313**	**OR335876**	**OR287059**
	**p43 / SLF 0218.1002**	**OR335882**	**OR287065**
Emericellopsis atlantica L.W. Hou, Crous, Rämä & Hagestad	CBS 147198 T	OL539742.1	OL634966
Emericellopsis cladophorae M. Gonçalves, T. Vicente & A. Alves	CMG25 T	MK986711	MK984311
Emericellopsis donezkii Beliakova	CBS 489.71 T	NR_156195.1	AY632674.1
Emericellopsis enteromorphae M. Gonçalves, T. Vicente & A. Alves	CMG26 T	MK986712	MK984312
Emericellopsis glabra (J.F.H. Beyma) Backus & Orpurt	CBS 119.40 T	NR_145024.1	AY632673.1
Emericellopsis humicola (Cain) Cain ex Grosklags & Swift	CBS 180.56 T	NR_145025.1	AY632675.1
Emericellopsis koreana Hyang B. Lee, S.J. Jeon & T.T.T. Nguyen	CNUFC-MOG1-1 T	MH173304	MH243035
Emericellopsis maritima Beliakova	CBS 491.71 T	KC987175	KC987137
Emericellopsis microspora Backus & Orpurt	CBS 380.62 T	NR_156196.1	AY632679.1
Emericellopsis minima Stolk	CBS 190.55 T	KC987173	KC987135
Emericellopsis mirabilis (Malan) Stolk	CBS 177.53 T	AY632656	-
	NBRC 114971	MW595829	LC605902
Emericellopsis pallida Beliakova	CBS 490.71 T	KC987176	KC987138
Emericellopsis persica Papizadeh, Wijayaw., Soudi & K.D. Hyde	IBRC-M 30046 T	KX668543	-
Emericellopsis phycophila M. Gonçalves, T. Vicente & A. Alves	CMG15 T	MK986701	MK984301

Emericellopsis pusilla P.N. Mathur, Sukapure & Thirum.	CBS 226.62 T	MH858143	-
Emericellopsis robusta Emden & W. Gams	CBS 105.70 T	MH859510	-
	CBS 489.73	AY632664	AY632680
*Emericellopsis salmonea (*W. Gams & Lodha) L.W. Hou, L. Cai & Crous	CBS 721.71 T	MH860309	-
Emericellopsis salmosynnemata Grosklags & Swift	CBS 182.56 T	MH857571.1	-
Emericellopsis stolkiae D.E. Davidson & M. Chr.	CBS 159.71 T	NR_156197.1	AY632684.1
Emericellopsis terricola J.F.H. Beyma	CBS 120.40 T	U57676	-
	CBS 229.59	AY632662	AY632678
Emericellopsis brunneiguttula L.W. Hou, L. Cai & Crous	CBS 111360 T	OQ429545	AY632689
Emericellopsis exuviara (Sigler, Zuccaro, Summerbell & Paré) L.W. Hou, L. Cai & Crous	CBS 113360 T	AY882946	AY882947
Emericellopsis fimetaria (Pers.) L.W. Hou, L. Cai & Crous	CBS 176.60	AY632665.1	AY632681.1
	CBS 382.62	AY632666	AY632682
	CBS 117.84	MH861705.1	-
	CBS 628.85	MH861906.1	-
	**p24 / SLF 0218.0620**	**OR335869**	**OR287052**
	**p26 / SLF 0218.0308**	**OR335871**	**OR287054**
	**p29 / SLF 0218.0609**	**OR335873**	**OR287056**
Emericellopsis fuci (Summerbell, Zuccaro & W. Gams) L.W. Hou, L. Cai & Crous	CBS 112868 T	AY632653	AY632690.1
Emericellopsis moniliformis (A. Giraldo, Deanna A. Sutton & Guarro) L.W. Hou, L. Cai & Crous	CBS 139051 T	LN810516	LN810523
Emericellopsis tubakii (Gams) L.W. Hou, L. Cai & Crous	CBS 790.69 T	MH859429	-
Emericellopsis sp.	A104	KC987141	KC987103
Emericellopsis sp.	A105	KC987142	KC987104
Emericellopsis sp.	A106	KC987143	KC987105
Emericellopsis sp.	A107	KC987144	KC987106
Emericellopsis sp.	A108	KC987145	KC987107
Emericellopsis sp.	A110	KC987147	KC987109
Emericellopsis sp.	A111	KC987148	KC987110
Emericellopsis sp.	E102	KC987172	KC987134
**Emericellopsis sp.**	**p20 / SLF 0218.0117**	**OR335866**	**OR287049**
**Emericellopsis sp.**	**p21 / SLF 0218.0708**	**OR335867**	**OR287050**
**Emericellopsis sp.**	**p22 / SLF 0218.0101**	**OR335868**	**OR287051**
**Emericellopsis sp.**	**p25 / SLF 0218.0601**	**OR335870**	**OR287053**
**Emericellopsis sp.**	**p27 / SLF 0218.0504**	**OR335872**	**OR287055**
**Emericellopsis sp.**	**p32 / SLF 0218.0908**	**OR335875**	**OR287058**
**Emericellopsis sp.**	**p37 / SLF 0218.0401**	**OR335877**	**OR287060**
**Emericellopsis sp.**	**p38 / SLF 0218.0203**	**OR335878**	**OR287061**
**Emericellopsis sp.**	**p39 / SLF 0218.0701**	**OR335879**	**OR287062**
**Emericellopsis sp.**	**p40 / SLF 0218.0702**	**OR335880**	**OR287063**
**Emericellopsis sp.**	**p41 / SLF 0218.0312**	**OR335881**	**OR287064**
**Emericellopsis sp.**	**p45 / SLF 0218.0201**	**OR335883**	**OR287066**
**Emericellopsis sp.**	**p46 / SLF 0218.0801**	**OR335884**	**OR287067**
**Emericellopsis sp.**	**p49 / SLF 0218.1006**	**OR335885**	**OR287068**
Furcasterigmium furcatum (C. Moreau & Moreau ex W. Gams) Giraldo López & Crous	CBS 122.42 T	AY378154	-
Fusarium algeriense Laraba & O'Donnell	CBS 142638 T	NR_158423.1	-
Fusarium beomiforme P.E. Nelson, Toussoun & L.W. Burgess	CBS 100160 T	MH862691.1	-
	CBS 740.97	U61674	-
Fusarium breve (Sand.-Den. & Crous) O'Donnell, Geiser, Kasson & T. Aoki	CBS 144387 T	LR583708	-

(= *Neocosmospora brevis* Sand.-Den. & Crous)			
Fusarium burgessii M.H. Laurence, Summerell & E.C.Y. Liew	CBS 125537 T	NR_172292.1	-
Fusarium equiseti (Corda) Sacc.	CBS 307.94 NT	MH862468.1	-
Fusarium incarnatum (Desm.) Sacc.	CBS 161.25 T	MH854830.1	-
Fusarium mori (Sand.-Den. & Crous) O'Donnell, Geiser, Kasson & T. Aoki (= *Neocosmospora mori* Sand.-Den. & Crous)	CBS 145467 T	DQ094305	-
Fusarium neerlandicum (Crous & Sand.-Den.) T. Aoki, Geiser & O'Donnell (= *Neocosmospora neerlandica* Crous & Sand.-Den.)	CBS 232.34 T	MH855496.1	-
Fusarium paulenelsonii T. Aoki, Geiser & O'Donnell (= *Neocosmospora nelsonii* Crous & Sand.-Den.)	CBS 309.75 T	MW827630	-
Fusarium pseudopisi (Sand.-Den. & L. Lombard) T. Aoki, Geiser & O'Donnell (= *Neocosmospora pseudopisi* Sand.-Den. & L. Lombard)	CBS 266.50 T	MH856618.1	-
Fusarium quercinum O'Donnell, Geiser, Kasson & T. Aoki (= *Neocosmospora quercicola* Sand.-Den. & Crous)	CBS 141.90 T	LR583760	-
Fusarium regulare (Sand.-Den. & Crous) O'Donnell, Geiser, Kasson & T. Aoki (= *Neocosmospora regularis* Sand.-Den. & Crous)	CBS 230.34 T	LR583763	-
	CBS 190.35	LR583762	-
Fusarium silvicola (Sand.-Den. & Crous) O'Donnell, Geiser, Kasson & T. Aoki (= *Neocosmospora silvicola* Sand.-Den. & Crous)	CBS 123846 T	LR583766	-
Fusarium solani (Mart.) Sacc. (= *Neocosmospora solani* (Mart.) L. Lombard & Crous)	CBS 140079 ET	KT313633	-
Fusarium solani f. *pisi* (F.R. Jones) W.C. Snyder & H.N. Hansen (= *Neocosmospora pisi* (F.R. Jones) Sand.-Denis & Crous)	CBS 181.29	MH855035.1	-
	CBS 123669	LR583753	-
	CBS 127118	MH864425.1	-
	CBS 188.34	MH855484.1	-
***Fusarium* sp. complex burgessii**	**p28 / SLF 0218.0204**	**OR335857**	-
***Fusarium* sp. complex incarnatum-equiseti**	**p7 / SLF 0218.0106**	**OR335853**	-
***Fusarium* sp. complex incarnatum-equiseti**	**p12 / SLF 0218.0404**	**OR335855**	-
***Fusarium* sp. complex solani**	**p9 / SLF 0218.0309**	**OR335854**	-
***Fusarium* sp. complex solani**	**p13 / SLF 0218.0405**	**OR335856**	-
Gibellulopsis aquatica Giraldo López & Crous	CBS 117131 T	LR026720	-
Gibellulopsis catenata Giraldo López & Crous	CBS 113951 T	LR026721	-
Gibellulopsis fusca (Thirum. & Sukapure) Giraldo Lopez & Crous	CBS 560.65 T	LR026724	-
Gibellulopsis nigrescens (Pethybr.) Zare, W. Gams & Summerb.	CBS 120949 NT	LR026738	-
	CBS 179.40	LR026727	-
	**p17 / SLF 0218.0103**	**OR335859**	-
Gibellulopsis serrae (Maffei) Giraldo Lopez & Crous	CBS 290.30 T	LR026742	-
	CBS 387.35	LR026745	-
	CBS 892.70	LR026755	-
	**p8 / SLF 0218.0302**	**OR335858**	-
***Myriodontium keratinophilum* Samson & Polon.**	**p15 / SLF 0218.0510**	**OR335852**	-
Penicillium allii-sativi Frisvad, Houbraken & Samson	CBS 132074 T	JX997021	-
Penicillium bialowiezense K.M. Zalessky	CBS 227.28 T	NR_165994.1	-
Penicillium brevicompactum Dierckx	CBS 257.29 T	KF465776.1	-
Penicillium chrysogenum Thom	CBS 306.48 T	MH856357.1	-
Penicillium confertum (Frisvad, Filt. & Wicklow)	CBS 171.87 T	JX997081	-

Frisvad			
Penicillium copticola Houbraken, Frisvad & Samson	CBS 127355 T	JN617685	-
Penicillium desertorum Frisvad, Houbraken & Samson	CBS 131543 T	JX997011	-
Penicillium dipodomyus (Frisvad, Filt. & Wicklow) Banke, Frisvad & S. Rosend.	CBS 110412 T	MH862862.1	-
Penicillium dokdoense Hyang B. Lee & T.T.T. Nguyen	JMRC:SF:013606 T	MG906868	-
Penicillium fennelliae Stolk	CBS 711.68 T	JX313169	-
Penicillium flavigenum Frisvad & Samson	CBS 419.89 T	MH862182.1	-
Penicillium halotolerans Frisvad, Houbraken & Samson	CBS 131537 T	JX997005	-
Penicillium kongii Long Wang	HMAS 244382 T	KC427191	-
Penicillium mononematosum (Frisvad, Filt. & Wicklow) Frisvad	CBS 172.87 T	JX997082	-
Penicillium nalgiovense Laxa	CBS 352.48 T	MH856385.1	-
Penicillium neocrassum R. Serra & S.W. Peterson	CBS 122428 T	DQ645805	-
Penicillium rubens Biourge	CBS 129667 T	NR_111815.1	-
Penicillium tardochrysogenum Frisvad, Houbraken & Samson	CBS 132200 T	JX997028	-
Penicillium terrigenum Houbraken, Frisvad & Samson	CBS 127354 T	NR_121515.1	-
Penicillium vanluykii Frisvad, Houbraken & Samson	CBS 131539 T	JX997007	-
Penicillium* sp. sect. *Brevicompacta* ser. *Brevicompacta	**p302 / SLF 0218.0562**	**OR335841**	-
Penicillium* sp. sect. *Brevicompacta* ser. *Brevicompacta	**p48 / SLF 0218.1005**	**OR335845**	-
Penicillium* sp. sect. *Chrysogena* ser. *Chrysogena	**p2 / SLF 0218.0902**	**OR335839**	-
Penicillium* sp. sect. *Chrysogena* ser. *Chrysogena	**p301 / SLF 0218.0561**	**OR335840**	-
Penicillium* sp. sect. *Chrysogena* ser. *Chrysogena	**p4 / SLF 0218.0915**	**OR335842**	-
Penicillium* sp. sect. *Chrysogena* ser. *Chrysogena	**p5 / SLF 0218.0903**	**OR335843**	-
Penicillium* sp. sect. *Chrysogena* ser. *Chrysogena	**p6 / SLF 0218.0802**	**OR335844**	-
Penicillium* sp. sect. *Citrina* ser. *Copticolarum	**p1 / SLF 0218.0407**	**OR335838**	-
Plectosphaerella cucumerina (Lindf.) W. Gams	CBS 137.37 T	MH855856	-
Pseudeurotium bakeri C. Booth	CBS 878.71 T	NR145345	-
	CBS 128112	MH864832.1	-
	**p47 / SLF 0218.1004**	**OR335863**	-
Pseudeurotium desertorum Mouchacca	CBS 986.72 T	JX076946	-
Pseudeurotium hygrophilum (Sogonov, W. Gams, Summerb. & Schroers) Minnis & D.L. Lindner	CBS 102670 T	NR_111128.1	-
Pseudeurotium ovale Stolk	CBS 389.54 T	NR_145346.1	-
Pseudeurotium zonatum J.F.H. Beyma	CBS 329.36 T	NR111127	-
Stanjemonium grisellum W. Gams, Schroers & M. Christensen	CBS 655.79 T	AY632671	AY632687.1
Thelebolus globosus Brummelen & de Hoog	CBS 113940 T	MH862951	-

T - ex-type strains, NT – neotype strains. Accession numbers of sequences generated in this study are in bold.

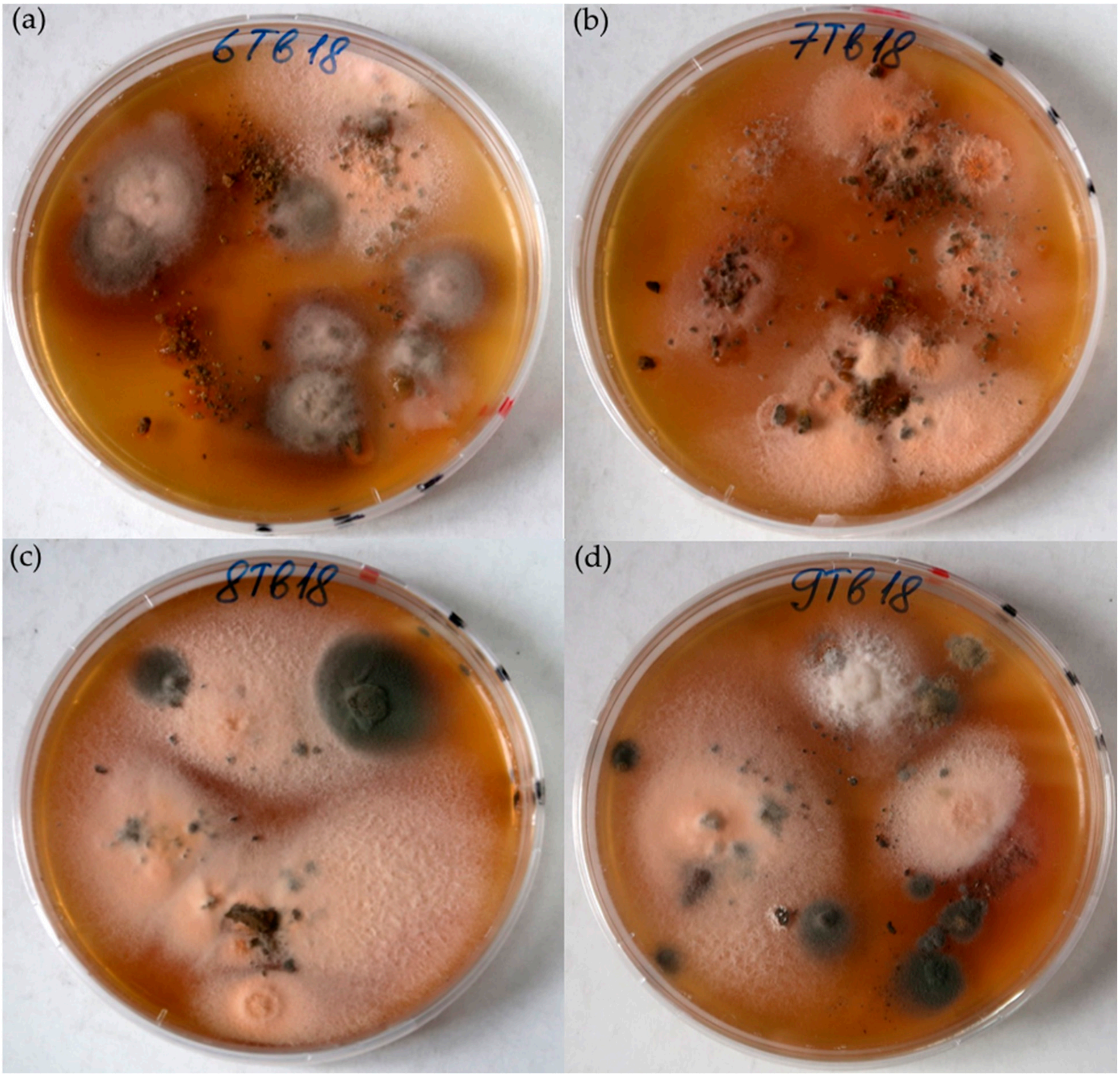

Figure S1. Samples of sediments of the Big Tambukan Lake on AA medium after 14 days of incubation: **a** – sample No 6; **b** – sample No 7; **c** – sample No 8; **d** – sample No 9.

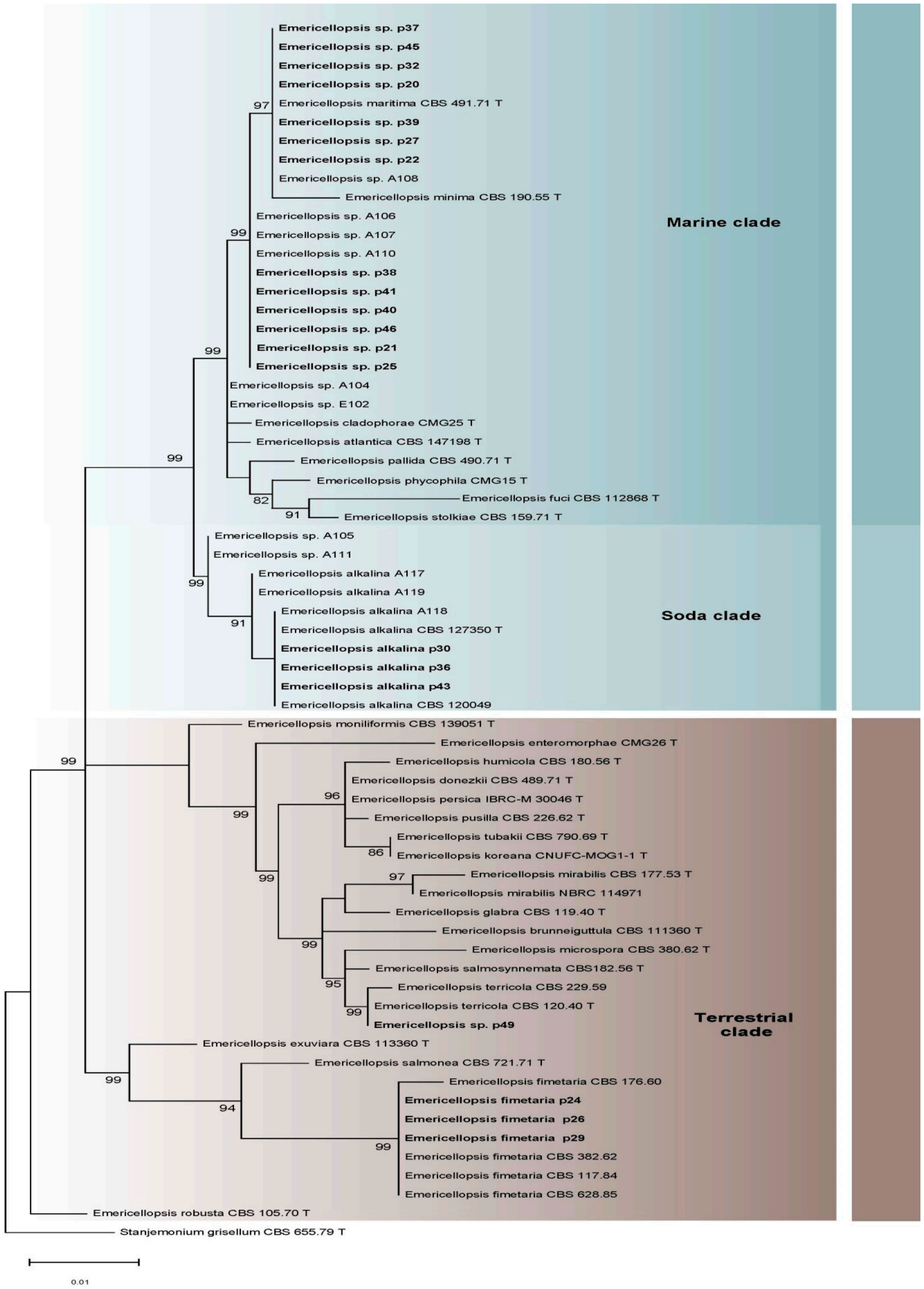

Figure S2. Maximum likelihood tree for the *Emericellopsis* genus based on partial sequences for the ITS rDNA (including 5.8S rDNA) region. Branch lengths are proportional to the estimated number of nucleotide substitutions. The BP values are displayed on the nodes (BP; 1000 replicates). *Emericellopsis* spp. and related species were clustered into a "Marine", "Soda", or "Terrestrial" clade. Taxa names of the isolates obtained in this study are in bold. "T" beside each strain name indicates the strains as the ex-type strain.

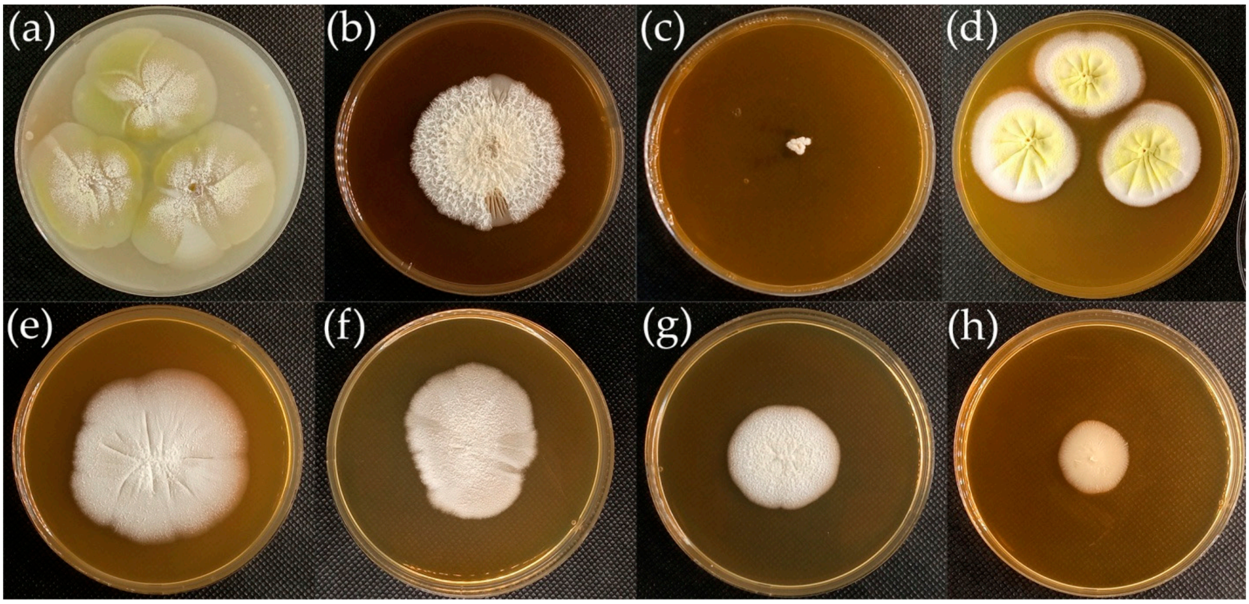

Figure S3. *Emericellopsis fimetaria* p24, 14 days-old (25 °C, dark regime) colonies in 9 cm Petri plates: **a** – on OA; **b** – on MYA; **c** – on MYA-based medium buffered at pH 10; **d** – 45 days-old colony on AA; **e–h** – on MYA containing 2.5%, 5%, 7.5% and 10% respectively (w/v) NaCl.

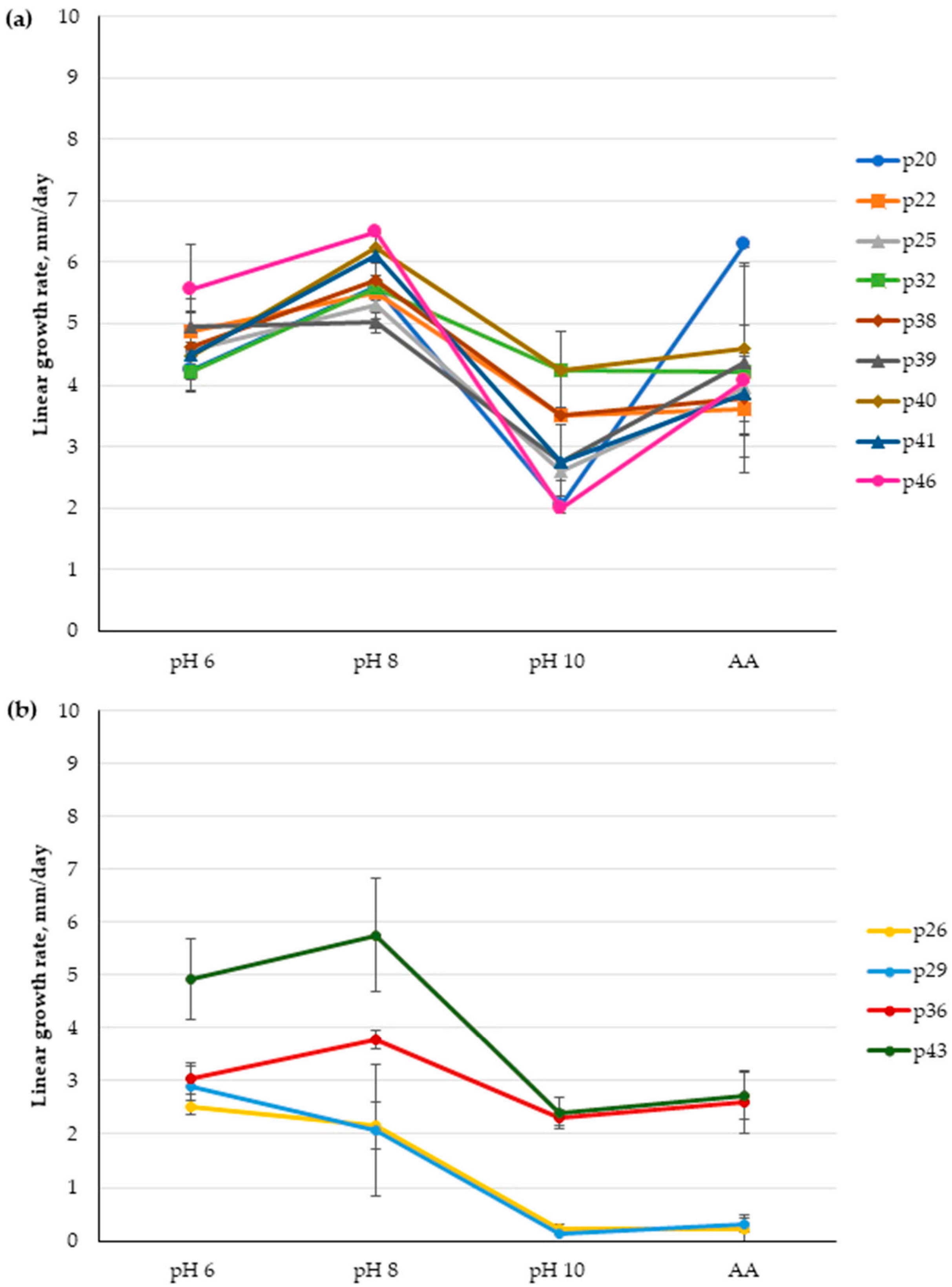

Figure S4. Linear growth rate patterns of *Emericellopsis* strains at different pH values. **a** - Marine clade: *Emericellopsis* sp. (p20, p22, p25, p32, p38, p39, p40, p41, p46); **b** - Terrestrial clade: *E. fimetaria* (p26, p29) and Soda clade: *E. alkalina* (p36, p43) (n=4, α=0.05, mean± CI).

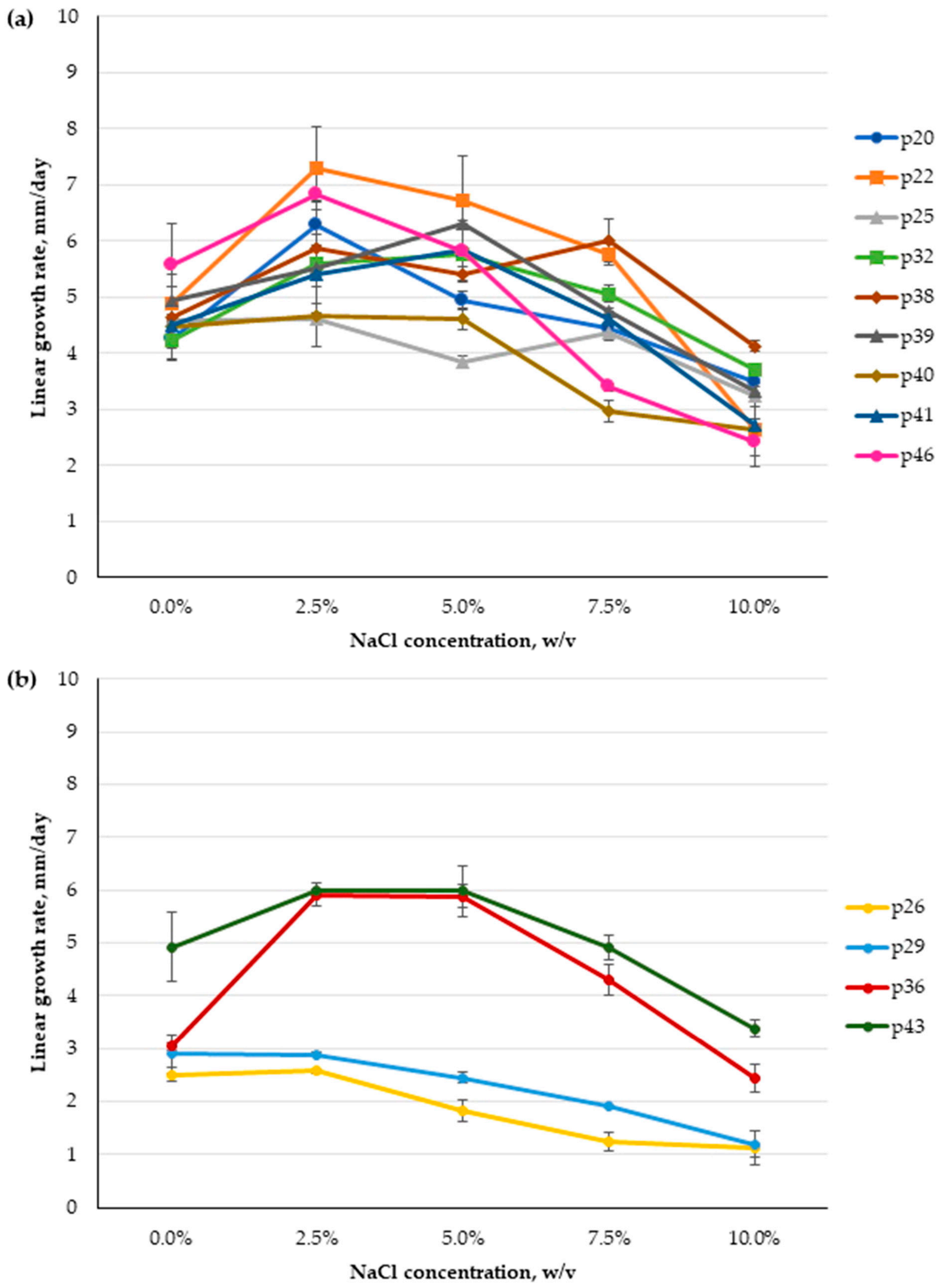

Figure S5. Linear growth rate patterns of *Emericellopsis* strains at different NaCl concentrations. **a** - Marine clade: *Emericellopsis* sp. (p20, p22, p25, p32, p38, p39, p40, p41, p46); **b** - Terrestrial clade: *E. fimetaria* (p26, p29) and Soda clade: *E. alkalina* (p36, p43) (n=4, α=0.05, mean± CI).

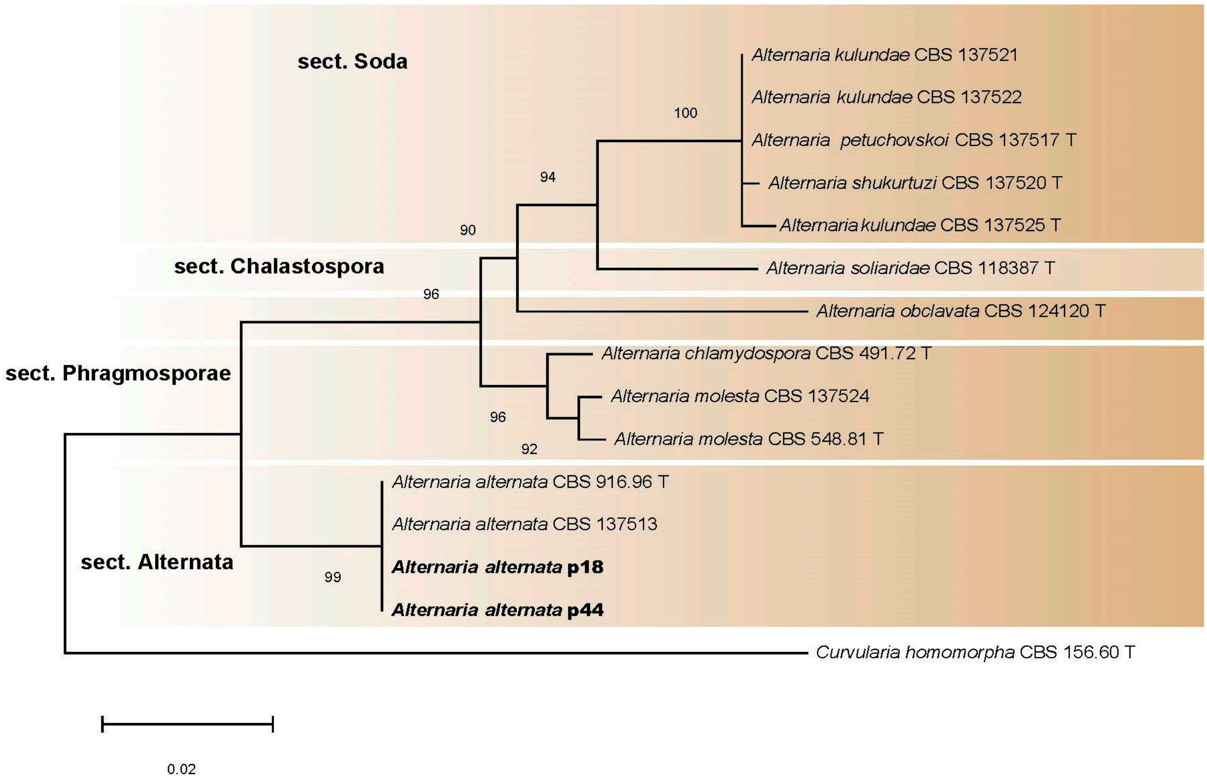

Figure S6. Phylogeny of *Alternaria alternata* isolates sequences (marked in bold) and closely related species based on ITS rDNA. ML support values are displayed over each node. T – ex-type strains.

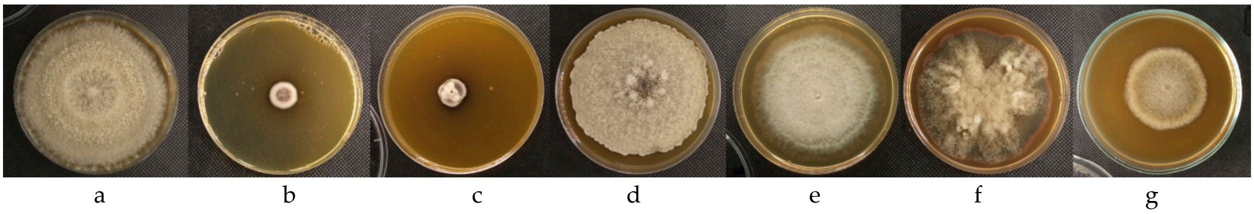

Figure S7. *Alternaria alternata*. **a–g** 14 days-old (25°C, dark regime) colonies in 9 cm Petri plates (isolate p44): **a** – on MYA medium (pH 6; 0% NaCl); **b** – on MYA-based media buffered at pH 10; **c** – on AA; **d–g** – on MYA containing 2.5%, 5%, 7.5% and 10% respectively (w/v) NaCl.

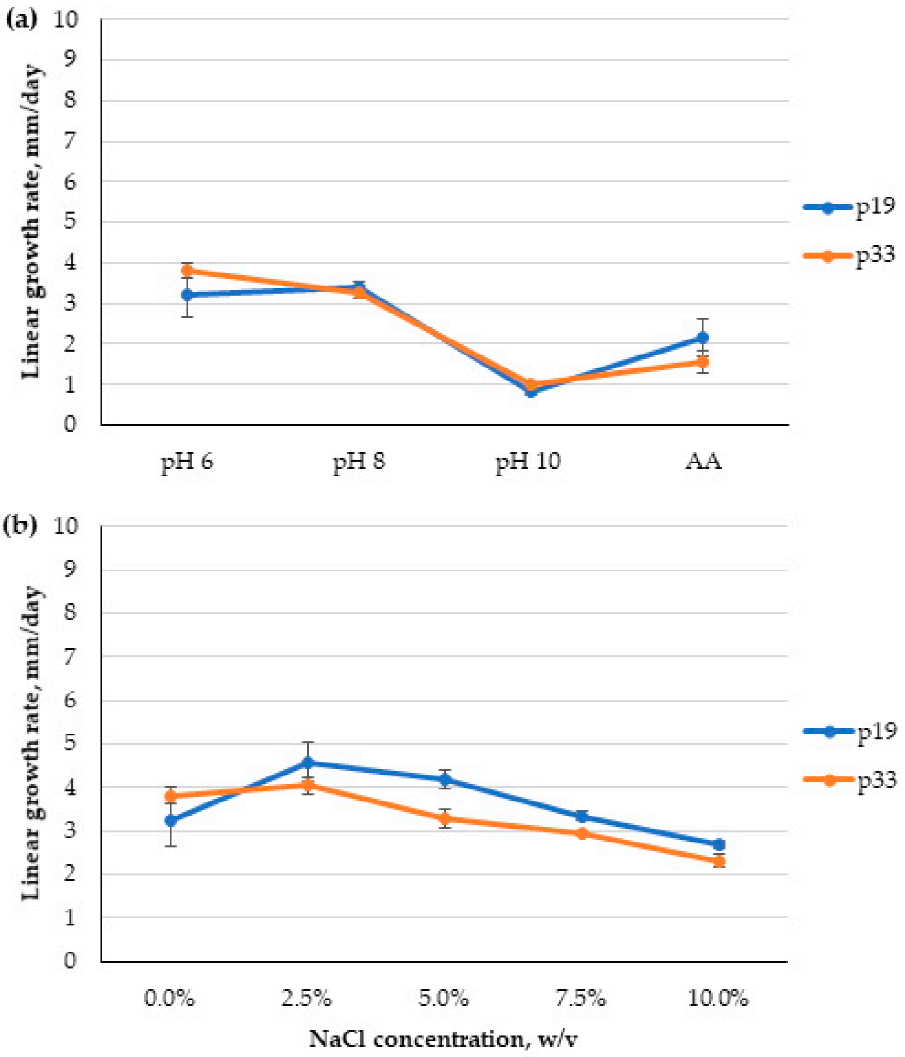

Figure S8. Linear growth rate patterns of *Acremonium egyptiacum* (p19 and p33): **a** – at different pH values; **b** – at different NaCl concentrations (n=4, α=0.05, mean± CI).

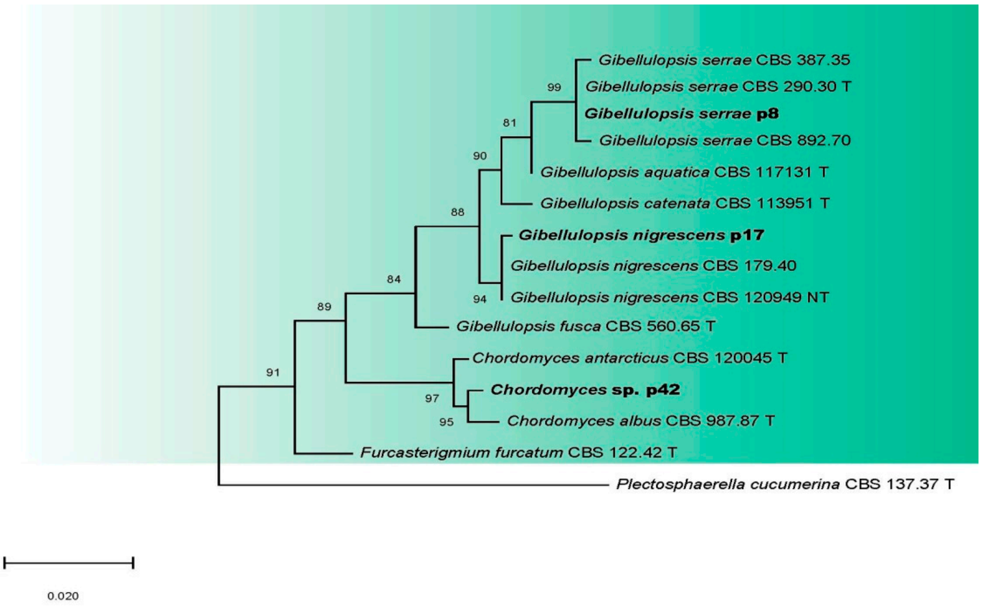

Figure S9. Phylogenetic analysis of Plectosphaerellaceae (*Gibellulopsis* and *Chordomyces*) isolates (marked in bold) based on ITS rDNA sequences. ML support values are displayed over each node. T – ex-type strains; NT – neotype strain.

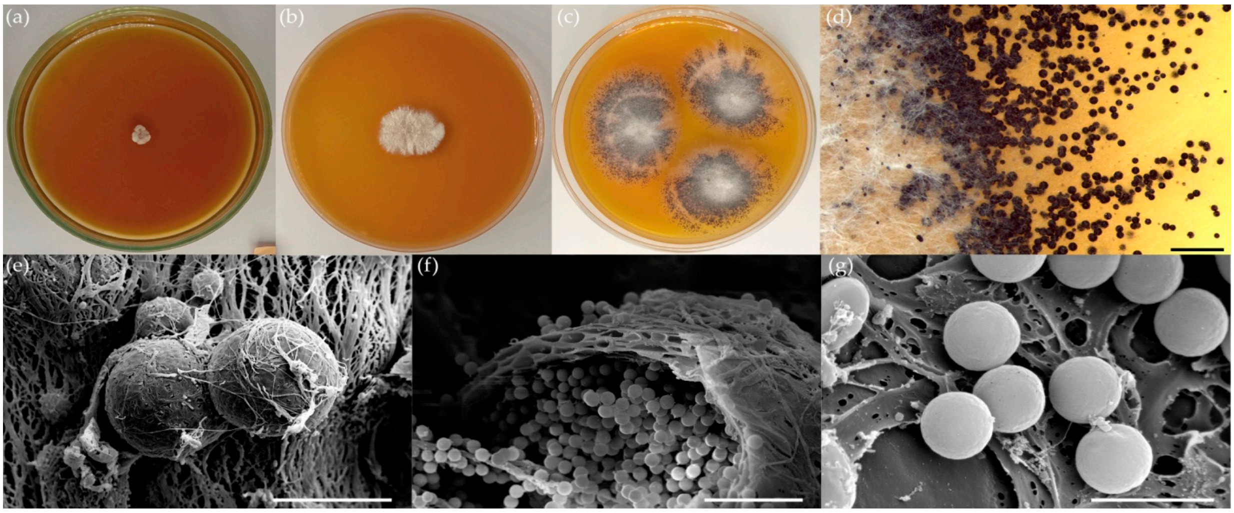

Figure S10. *Pseudeurotium bakeri* (p47). **a–c** 21 days-old (25 °C, dark regime) colonies in 9 cm Petri plates: **a–b** – on MYA containing 5% and 2.5% respectively (w/v) NaCl; **c** – on MYA (0% NaCl, pH 6); **d** – colony margin at MYA with enclosed fruiting bodies (LM); **e** – ascomata fruiting bodies (SEM); **f** – broken asomata cleistothecium with ascospores, note multilayered wall (SEM); **g** – ascospores (SEM). Scale bars: d = 1 mm; e = 100 µm; f = 20 µm; g = 5 µm.

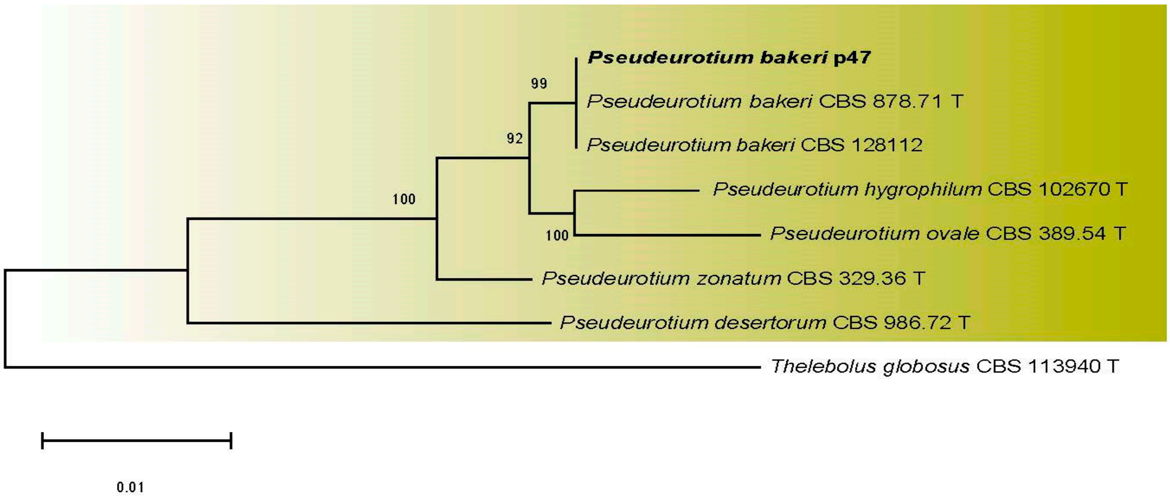

Figure S11. ITS rDNA-based phylogeny of the obtained *Pseudeurotium bakeri* strain p47 (marked in bold) and closely related species. ML support values are displayed over each node. T – ex-type strains.

Table S2. Antimicrobial activity of *Emericellopsis* genus

Strain No	Species	Medium	Zone of inhibition, mm			
			E.coli ATCC 25922	*B.subtilis* ATCC 6633	*A.niger* INA 00760	*C.albicans* ATCC 2091
p20	*Emericellopsis* sp. Marine clade	AA	0	12±0.1	22±0.3	32±0.2
		MYA	0	13±0.2	0	0
		CZA	0	22±0.1	10±0.2	0
p21	*Emericellopsis* sp. Marine clade	AA	0	13±0.1	27±0.2	26±0.4
		MYA	10±0.1	16±0.1	0	10±0.2
		CZA	17±0.3	25±0.1	0	14±0.2
p22	*Emericellopsis* sp. Marine clade	AA	0	12±0.1	23±0.3	25±0.1
		MYA	0	15±0.2	0	0
		CZA	0	22±0.2	9±0.2	0
p24	*Emericellopsis fimetaria*	AA	0	0	0	0
		MYA	0	14±0.1	15±0.3	12±0.2
		CZA	0	18±0.2	13±0.1	0
p25	*Emericellopsis* sp. Marine clade	AA	0	0	0	0
		MYA	0	18±0.4	0	0
		CZA	13±0.2	16±0.1	0	0
p26	*Emericellopsis fimetaria*	AA	0	0	0	0
		MYA	0	18±0.2	0	20±0.2
		CZA	0	11±0.3	17±0.3	17±0.2
p27	*Emericellopsis* sp. Marine clade	AA	0	15±0.1	22±0.1	10±0.2
		MYA	0	16±0.1	10±0.3	0
		CZA	0	22±0.3	10±0.3	0
p29	*Emericellopsis fimetaria*	AA	0	0	0	0
		MYA	0	11±0.2	16±0.3	12±0.2
		CZA	0	11±0.2	0	0
p30	*Emericellopsis alkalina*	AA	0	14±0.1	25±0.3	25±0.4
		MYA	0	0	0	0
		CZA	14±0.2	0	0	0
p32	*Emericellopsis* sp. Marine clade	AA	0	12±0.3	26±0.2	25±0.3
		MYA	0	11±0.1	0	9±0.1
		CZA	9±0.2	16±0.3	0	13±0.4
p36	*Emericellopsis alkalina*	AA	0	12±0.1	24±0.3	22±0.4
		MYA	9±0.2	0	9±0.2	0
		CZA	20±0.3	0	12±0.2	0
p37	*Emericellopsis* sp. Marine clade	AA	0	13±0.1	22±0.1	10±0.1

		MYA	10±0.3	21±0.2	0	0
		CZA	12±0.1	24±0.2	0	0
p38	*Emericellopsis* sp. Marine clade	AA	0	11±0.2	21±0.4	19±0.3
		MYA	0	16±0.1	0	0
		CZA	14±0.2	24±0.2	0	0
p39	*Emericellopsis* sp. Marine clade	AA	0	21±0.2	23±0.2	25±0.3
		MYA	0	14±0.2	0	0
		CZA	13±0.1	18±0.2	0	0
p40	*Emericellopsis* sp. Marine clade	AA	0	12±0.4	23±0.2	30±0.3
		MYA	0	12±0.2	0	0
		CZA	11±0.1	18±0.1	0	9±0.1
p41	*Emericellopsis* sp. Marine clade	AA	9±0.2	13±0.3	27±0.3	28±0.2
		MYA	0	17±0.2	9±0.1	0
		CZA	16±0.1	22±0.2	10±0.2	0
p43	*Emericellopsis alkalina*	AA	0	0	0	0
		MYA	0	15±0.3	9±0.1	0
		CZA	0	17±0.2	0	0
p45	*Emericellopsis* sp. Marine clade	AA	0	0	0	0
		MYA	10±0.1	13±0.1	0	0
		CZA	14±0.3	15±0.2	16±0.1	0
p46	*Emericellopsis* sp. Marine clade	AA	0	14±0.1	23±0.2	22±0.3
		MYA	9±0.1	15±0.2	11±0.1	10±0.1
		CZA	9±0.1	15±0.2	9±0.1	15±0.3
p49	*Emericellopsis* sp. Terrestrial clade	AA	0	0	0	0
		MYA	9±0.1	0	0	0
		CZA	0	0	0	0

Disk diameter – 6 mm

Table S3. Antimicrobial activity of other genera obtained.

Strain No	Species	Medium	Zone of inhibition, mm			
			E.coli **ATCC 25922**	***B.subtilis*** **ATCC 6633**	***A.niger*** **INA 00760**	***C.albicans*** **ATCC 2091**
p1	*Penicillium* sp. sect. *Citrina* ser. *Copticolarum*	AA	0	0	0	0
		MYA	0	0	14±0.1	0
		CZA	0	9±0.2	0	0
p2	*Penicillium* sp. sect. *Chrysogena* ser. *Chrysogena*	AA	0	0	0	0
		MYA	0	0	0	0
		CZA	0	0	0	9±0.2
p301	*Penicillium* sp. sect. *Chrysogena* ser. *Chrysogena*	AA	0	0	0	0
		MYA	0	0	0	0
		CZA	0	15±0.3	0	0

p302	*Penicillium* sp. sect. *Brevicompacta* ser. *Brevicompacta*	AA	0	0	0	0
		MYA	0	0	0	0
		CZA	0	15±0.2	0	0
p4	*Penicillium* sp. sect. *Chrysogena* ser. *Chrysogena*	AA	0	0	0	0
		MYA	0	9±0.1	0	0
		CZA	0	9±0.1	9±0.2	0
p5	*Penicillium* sp. sect. *Chrysogena* ser. *Chrysogena*	AA	0	0	0	0
		MYA	10±0.2	10±0.2	9±0.1	0
		CZA	0	0	0	0
p6	*Penicillium* sp. sect. *Chrysogena* ser. *Chrysogena*	AA	0	0	0	0
		MYA	0	9±0.1	0	0
		CZA	0	16±0.3	0	0
p48	*Penicillium* sp. sect. *Brevicompacta* ser. *Brevicompacta*	AA	0	0	0	0
		MYA	11±0.2	0	0	9±0.2
		CZA	0	0	0	0
p10	*Aspergillus* sp. sect. *Nidulantes* ser. *Versicolores*	AA	0	0	0	0
		MYA	0	0	0	0
		CZA	0	0	0	0
p11	*Aspergillus* sp. sect. *Flavipedes* ser. *Spelaei*	AA	0	0	0	0
		MYA	0	11±0.1	0	10±0.2
		CZA	0	9±0.2	10±0.3	9±0.2
p14	*Aspergillus* sp. sect. *Flavipedes* ser. *Flavipedes*	AA	0	0	0	0
		MYA	0	0	0	0
		CZA	9±0.2	15±0.3	10±0.1	14±0.2
p16	*Aspergillus* sp. sect. *Nidulantes* ser. *Versicolores*	AA	0	0	0	0
		MYA	0	0	0	0
		CZA	0	9±0.1	0	0
p34	*Aspergillus* sp. sect. *Flavipedes* ser. *Spelaei*	AA	0	0	0	0
		MYA	0	13±0.3	13±0.2	12±0.2
		CZA	17±0.3	0	12±0.2	0
p35	*Aspergillus* sp. sect. *Flavipedes* ser. *Flavipedes*	AA	0	0	0	0
		MYA	0	18±0.3	0	9±0.1
		CZA	0	18±0.2	0	9±0.2
p7	*Fusarium* sp. complex incarnatum-equiseti	AA	0	0	0	0
		MYA	0	18±0.2	0	19±0.3
		CZA	0	17±0.2	0	16±0.2
p9	*Fusarium* sp. complex solani	AA	0	0	0	0
		MYA	0	0	0	0
		CZA	0	9±0.2	10±0.2	0

p12	*Fusarium* sp. complex incarnatum-equiseti	AA	0	0	0	0
		MYA	0	15±0.1	16±0.1	15±0.3
		CZA	0	12±0.2	0	14±0.2
p13	*Fusarium* sp. complex solani	AA	0	0	0	0
		MYA	0	0	0	0
		CZA	0	0	0	0
p28	*Fusarium* sp. complex burgessii	AA	0	0	0	0
		MYA	0	13±0.3	17±0.1	0
		CZA	0	14±0.1	17±0.2	0
p8	*Gibellulopsis serrae*	AA	0	0	0	0
		MYA	0	0	0	0
		CZA	0	0	0	0
p17	*Gibellulopsis nigrescens*	AA	0	0	0	0
		MYA	0	0	0	0
		CZA	10±0.2	9±0.1	9±0.1	0
p18	*Alternaria alternata*	AA	0	0	0	0
		MYA	0	15±0.3	14±0.2	0
		CZA	0	0	0	11±0.2
p44	*Alternaria alternata*	AA	0	0	0	0
		MYA	0	12±0.1	0	9±0.2
		CZA	0	0	13±0.3	9±0.2
p19	*Acremonium egyptiacum*	AA	0	12±0.1	23±0.2	28±0.3
		MYA	0	11±0.1	9±0.2	10±0.2
		CZA	18±0.3	0	9±0.1	0
p33	*Acremonium egyptiacum*	AA	0	0	0	0
		MYA	0	15±0.2	0	0
		CZA	0	0	0	0
p15	*Myriodontium keratinophilum*	AA	0	0	0	0
		MYA	0	14±0.1	0	14±0.2
		CZA	0	0	0	0
p47	*Pseudeurotium bakeri*	AA	0	0	0	0
		MYA	0	15±0.2	0	10±0.2
		CZA	0	0	0	0
p42	*Chordomyces* sp.	AA	0	13±0.2	23±0.3	17±0.1
		MYA	0	0	0	0
		CZA	0	0	0	0

Disk diameter – 6 mm
